# Having a Sense of Humor as a Virtue

**DOI:** 10.1007/s10790-022-09918-1

**Published:** 2022-11-28

**Authors:** Mark Alfano, Mandi Astola, Paula Urbanowicz

**Affiliations:** 1 Macquarie University, 42a High Street, Millers Point, NSW 2000 Australia; 2 Delft University of Technology, Jaffalaan 5, 2628 BX Delft, The Netherlands; 3 Osnabrück University, Osnabrück, Germany

## Introduction

During the pandemic, recession, and protests for social justice in 2020, comedian Sarah Cooper took to TikTok and Twitter, posting short videos of herself lipsyncing to audio of Donald Trump making inane and nonsensical comments about current events.^1^ Her videos have been viewed and shared millions of times, and then-President Trump blocked her account on Twitter. In an interview on National Public Radio, Cooper said that creating these videos helped her “cope with anxiety, uncertainty and hopelessness.” She also reported that people had told her that her videos were the only thing that made them laugh in those difficult times, which suggests that her efforts at coping also helped others to cope. Reflecting on the impact she’d had, Cooper said, “I realize humor is important. Laughing is important. It’s healing. [...] And so as hard as everything is right now, I do think that there is something to be said for making things that people can have some release with.”^2^

In the same vein, in a recent interview, Ukrainian President Volodymyr Zelenskiy displayed irony and sarcasm; when asked about this, he told the reporter, “I think that any normal person cannot survive” without a sense of humor, and that “You can’t be serious about what Russian politicians and Lukashenko say every day. If you take it seriously, you might as well go and hang yourself.”^3^ He went on to say that a sense of humor is “a powerful weapon” that helps to reveal deep truths in an accessible way. In this paper, we explore these ideas at greater length and more systematically, arguing that having a sense of humor is a complex virtue that operates through the elicitation and expression of a range of emotions.

Here is the plan for this paper: in section 2, we review the philosophical literature connecting humor and virtue. In section 3, we discuss the connection between having a sense of humor and a range of emotions that humor both elicits and directs, namely amusement, contempt, trust, and hope. Next, in section 4, we draw on the role of emotions in humor to canvass several functions served by having a sense of humor. In particular, we argue that a sense of humor aids both its bearer and other people who are socially connected to the bearer with *coping, connection, capability*, and *criticism*. We contextualize this argument in several fields in which a sense of humor operates, namely hardship, social relationships, collective action, and existential reflection.

## The Philosophy of Humor

With the exception of some pathological cases involving schizophrenia and depression (Ivanova 2014; Parnowska 2013), almost everyone is disposed to laugh at or find humorous at least some things. In this minimal, descriptive sense, almost everyone has a sense of humor. However, it is also clear that there are normative dimensions to having a sense of humor. One’s sense of humor can be better or worse in multiple ways. For instance, in pathological cases of *Witzelsucht* (Granadillo and Mendez 2016), the patient exhibits a compulsion to make jokes, including both unfunny puns and attempts at sexual humor, and seems incapable of understanding negative feedback about such jokes. Those who suffer from *Witzelsucht* have a disposition to engage in humor too frequently, at the wrong time, in the wrong way, and for the wrong reasons.^4^ And, of course, there are many non-pathological cases of people who can’t tell or take a joke.^5^

The fact that we normatively assess someone’s sense of humor along these dimensions suggests that — like many of the more traditional virtues — it may be construed according to the Aristotelian doctrine of the mean (*Nicomachean Ethics* 1103a-1104b). Indeed, Aristotle held that *eutrapelia* (often translated as *wit*) is the mean between *bomolochos* (buffoonery) and *agroikos* (anhedonia) in the context of conversation (Arneson 2018). On this view, a virtuous sense of humor is a disposition that involves finding or making humor in or laughing at the right things, at the right time, in the right way, with the right people, for the right reason, with the right intensity, and so on. As we typically find with virtue-theoretic analyses, there is plenty of wiggle room and gray area here. It would be more precise to say that a virtuous sense of humor is a disposition that involves finding or making humor in or laughing at some of the right things, often enough at the right time, typically in the right way, usually with the right people, for some of the right reasons, with roughly the right intensity, and so on.

Specifying necessary and sufficient conditions for the virtuous exercise of a disposition is a fool’s errand, so we will not try to spell out exactly when and how a virtuous sense of humor should be manifested. A vicious sense of humor may be more obviously identifiable than a virtuous one. Someone embodies a vicious sense of humor to the extent that they are disposed to find or make humor in or laugh at (egregiously) the wrong things, at the wrong time, in the wrong way, with the wrong people, for the wrong reason, with the wrong intensity, and so on. This description includes cringy, cheugy, offensive, or annoying buffoonery, as well as the opposite vice, boring humorlessness or overseriousness. Morreall (2009) explores both the virtuous (chapter 6) and the vicious sides of the ethics of humor (chapter 5), arguing that these two polarities express and entrench the moral and intellectual virtues and vices, respectively, of those engaged in humor.

Furthermore, on the Aristotelian view, a virtuous sense of humor would have to be moderately reasons-responsive. Just as courage responds to reasons for confronting a threat or danger, so a virtuous sense of humor would have to respond to reasons for provoking amusement, finding something amusing, and laughing, but also to reasons for other emotional and behavioral reactions such as trusting the humorous person and others who are in on the joke. One might find this constraint too restrictive. After all, isn’t much humor responsive to *nonsense*, which is the opposite of reason? At the same time, it’s not incoherent to ask someone why they’re laughing or what’s so funny. People clearly expect each other to be able to explain their laughter and amusement at least sometimes and to some extent. We return to this connection between humor and fitting emotions in the next section.

Finally, in the Aristotelian framework, a virtuous sense of humor would have to be the sort of thing one can acquire through habituation.^6^ For instance, one might start off by catching infectious laughter from others who already have a well-developed sense of humor. Then one might learn some good jokes by rote, perhaps memorizing them from a book of jokes or by appreciating exemplars such as standup comics. Eventually, one’s sense of humor would be fine-tuned enough to function spontaneously and creatively. In this way, the acolyte of humor might learn when (and when not) to engage in humor of a particular sort in the company of some but not of others (Manke 1998; Olin 2022).^7^ If this developmental account is on the right track, it follows that if someone primarily has access only to those with a vicious sense of humor, they are liable to develop a vicious sense of humor themselves. Indeed, this seems to be an explicit recruitment strategy of the alt-right and other racist groups online (Greene 2019).

In light of these considerations, it’s plausible that having a sense of humor either is a virtue in its own right or depends critically on the possession of other dispositions that constitute virtues. This possibility has been raised in contemporary philosophy by several authors. For instance, Frankfurt (2001) suggests in passing that having a sense of humor may be especially important for people who see themselves as imperfect and who are therefore reluctant to take themselves too seriously. Frankfurt envisions a sense of humor as enabling one to move forward in life despite flaws and imperfections — not by ignoring them or pretending that they don’t exist, but by recognizing them and not putting too much weight on them, thus making a sense of humor a natural ally of humor and intellectual humility. Lippitt (2005) argues at greater length that a sense of humor is a virtue only when it is allied with further virtues. In addition, he argues that a sense of humor is valuable not only in the ethical domain but also in the epistemic domain. In particular, he thinks that a sense of humor often fosters self-recognition and self-knowledge. Deen (2018) also argues that a sense of humor is epistemically valuable, suggesting that it both engenders “an awareness of the limits of power” and promotes “a tendency not to take oneself too seriously.” Like Lippitt, Roberts (1988) thinks that a sense of humor is a virtue only when allied to other virtues, such as compassion and hope. When one’s sense of humor enjoys these partners, he argues, one is better able to laugh at oneself, which is essential to self-transcendence. Morton (2013, p. 133) also envisions an epistemic value to the ability to laugh. He argues that people who are incapable of laughter are not to be fully trusted because they have “no capacity to hold two points of view in mind.” Thus, a sense of humor may also help people to foster open-mindedness and the capacity for empathy. Gimbel (2018) argues that the capacity to engage in humor depends constitutively on intellectual virtues such as creativity and open-mindedness; he also suggests that sufficiently mean-spirited humor devoid of compassion and hope evinces bad moral character. And Morreall (2009) argues that a sense of humor fosters both moral and intellectual virtues such as open-mindedness and creativity, though it also presents the risk of fostering disengagement in ways that promote irresponsibility, cruelty, and prejudice.

Beyond philosophy, social scientists have found that a sense of humor is one of the most valued traits in initiating romantic relationships (Hall 2015), maintaining good relationships (Abel 1998), and remembering deceased loved ones (Alfano et al. 2018). These findings suggest that a sense of humor is a valued and valuable disposition. In this paper, we argue on functional emotional grounds that having a sense of humor is a virtue.

## A Sense of Humor’s Role in Emotion Expression, Elicitation, and Direction

It is a truism to say that emotions and virtues interact in complex ways. Aristotle connects many virtues with the emotion or emotions that they are meant to govern. For instance, he associates courage with the governance of fear (*Nicomachean Ethics* 1115a10-24). The courageous person often fears neither too much (which is what the coward typically does) nor too little (which is what a rash person typically does). Furthermore, the mapping from virtues to emotions need not be one-one. Aristotle also associates courage with the governance of confidence, which one can also be disposed to feel too much (an instance of rashness) or too little (an instance of cowardice). In addition, someone who embodies the virtue of compassion (rather than or in addition to the virtue of courage) might fear for the wellbeing of others. If this is right, then the mapping between virtues and emotions is neither one-one nor many-one but many-many (e.g., courage maps to multiple emotions, and multiple virtues map to fear). In this section, we argue that having a sense of humor exemplifies this complexity, and that it is especially closely tied to the emotions of amusement, contempt, trust, and hope.^8^ In particular, we suggest that sense of humor boosts emotions like amusement, contempt, trust and hope when they are deficient and inhibits them when they are excessive, bringing the emotion closer to the golden mean for each situation.

### Amusement

Perhaps the first emotion that springs to mind when one reflects on a sense of humor is amusement.^9^ A sense of humor both *enhances* and *inhibits* the emotion of amusement, in the sense that it enhances amusement in oneself and one’s audience, and inhibits amusement when a joke is deemed unfunny, too obscure, or offensive. Expressions of one’s sense of humor such as telling jokes, making puns, offering wry remarks, exchanging knowing glances, and so on are all meant to elicit amusement in another person. It’s a clear sign that someone’s sense of humor is poorly tuned if they are the only one laughing at their own jokes. Thus, a sense of humor is considerably more social than many traditional virtues such as courage. Whereas courage, in the first instance, governs fear and confidence in the courageous person herself, a sense of humor elicits amusement from socially connected others. This is not to suggest that a courageous person does not inspire confidence in others; indeed, that seems to be an important feature of courage and one of the reasons why it needs to be expressed in socially-recognizable ways. Nevertheless, many paradigm cases of courage are purely or primarily self-regarding. Having a sense of humor, by contrast, is primarily other-regarding. While it is no doubt possible to employ a sense of humor to help oneself laugh at and laugh off the troubles in one’s own life, humor is, in the first instance, social.

But what elicits amusement? The *benign violation* theory provides a plausible explanation of many paradigm cases. According to this theory, humor occurs when “(1) a circumstance is appraised as a violation, (2) the circumstance is appraised as benign, and (3) both appraisals occur simultaneously” (Warren and McGraw 2015, p. 75).^10^ In this theory, a violation is understood as anything that threatens or violates one’s normative beliefs. In other words, violations are everything that threatens one’s beliefs about *normality* or *norms*. These can be linguistic norms, social norms, cultural norms, logical norms, or moral norms. Even violations of one’s physical wellbeing fall under this heading. A benign violation is a violation that *seems* tolerable or safe. So, when someone perceives a violation and appraises it as benign at the same time, that person will likely experience amusement (McGraw and Warren 2010).

Being amused by harmless or safe violations may seem fitting, and amusement appears to be a fitting emotion in such cases.^11^ A good sense of humor therefore boosts amusement in these fitting cases. However, being amused by threats or violations that might actually be harmful or *are* already actively harmful seems unfitting. In unfitting cases, a good sense of humor inhibits amusement. This is expressed by not laughing, rolling one’s eyes, or directly telling someone they are not funny. We can get a clearer picture of the connections among violations, threats, humor, and amusement by thinking about dark humor, which is about or responsive to pain, suffering, human fragility and finitude, and evil. In other words, dark humor is about things that violate and threaten our normative beliefs about what a good and decent life is like: free from pain and suffering, without human fragility, finitude, and evil. How is it that people are able to experience amusement in connection with these phenomena? Why would it be fitting or appropriate to do so? One simple reason is that positive affect is an essential aspect of flourishing — an idea familiar from both hedonic and affective axiologies (Haybron 2008) and empirical psychology (Seligman 2011). Pain, suffering, fragility, finitude, and evil all make it difficult to enjoy positive affect. As the examples of Sarah Cooper’s videos and Zelenskiy’s sarcasm mentioned above show, a dose of (dark) humor in dark times can inject a bit of positive affect when and where it is most needed.

But again, this might seem to run counter to a fitting-attitudes approach to emotion. Amusement hardly seems fitting or appropriate in dark times. Indeed, persistent positive affect in response to suffering is a sign of pathological mania, not mental health. Would not sadness, anger, or despair be more appropriate in these contexts? This brings us to a useful distinction, articulated by Morton (2013; see also Alfano 2016, chapter 3), between two ways in which distinct emotions can interact in the same person. On the one hand, two emotions operating, as it were, at the same level might mix together directly. A relatively clear example is fright, which is an amalgam of fear and surprise. Someone who experiences fear of X and surprise at X at the same time could be said to experience fright at X. On the other hand, one emotion can take another emotion as its intentional object. Consider the case of a person who, unbeknownst to himself, has a phobia of spiders. He notices a spot on the wall, approaches it, and comes to see that it is a spider. In so doing, he is overcome with fear of the spider. But the spider hasn’t jumped out at him or appeared out of nowhere, so he is not surprised by the spider. Nevertheless, he is surprised *at his own fear* of the spider. This is a case not of fright but of recursively embedded emotions: he is surprised by his fear of the spider. Such recursive embedding can be reflexive (as it is in this case) or social. For instance, supposing our arachnophobe was otherwise fairly courageous, someone else might be surprised by his fear of the spider (without being surprised by the spider itself).

We suggest that the amusement involved in dark humor is often recursive rather than mixed. In other words, dark humor often involves finding something to laugh at in one’s own or other people’s suffering or pain or angst, rather than directly feeling amusement towards the source of suffering. For example, several amputees have had their remaining leg tattooed with, “I’m with stumpy ←.”^12^ In so doing, they make light of their own situation and perhaps help themselves cope with it. This explains how dark humor might be fitting or appropriate: dark humor makes light not of the source of suffering itself but of one’s own negative emotions towards suffering. In a bout of dark humor, one questions whether one’s own negative emotions are as worthy of concern as they appear from the inside, whether one’s own life is really as all-important as it feels to oneself. In so doing, dark humor can make negative affect loom less large than it otherwise would and furnish something approximating the perspective of the Spinozan *sub specie aeternitatis* or the laughing Epicurean gods.

McGraw and Warren conclude from their benign violation theory that “[l]aughter and amusement signal to the world that a violation is indeed okay” (McGraw and Warren 2010, p. 1148). This might sometimes be true, although it is hard to say what *indeed* is okay and what is not, and even harder in cases of dark humor. We suggest that a sense of humor often helps people to see *themselves* as okay (all things considered) rather than to perceive the violation itself as acceptable. This could explain why people with a sense of (dark) humor seem to be able to cope with suffering, pain, and evil.

### Contempt

Having a sense of humor is also closely associated with the emotion of contempt, which it also enhances when deficient and inhibits when excessive.^13^ We understand contempt broadly to involve a negative evaluation of the contemned person, disposition, action, or thing in comparison to better alternatives or a violated standard; contempt essentially involves looking *down* on someone or something. This does not mean that contempt is experienced negatively by the contemnor. Indeed, contempt often seems to involve positive affect, as it presupposes that the contemnor is *above* the object of contempt in some way. Like all emotions, contempt comes in degrees. The exact character of the negative evaluation can be absolute, judging the contemned to be totally without worth or importance; alternatively, it can be relative, judging the contemned to be *less* worthy or *less* important than it is taken to be without necessarily judging it to have no worth or importance at all.^14^

Furthermore, contempt motivates us to withdraw from the contemned person, disposition, or thing. Fisher and Roseman (2007), among others, argue that contempt has a different character from anger. In anger we are motivated to approach the person we are angry at and often aim for long-term reconciliation: anger may be met with guilt and contrition, which in turn may be met with forgiveness. When feeling contempt for X, however, we reject X and do our best to avoid or withdraw from it. Moreover, when X is a person or a group of people, contempt additionally motivates us to socially exclude this person or group. So anger and contempt have distinct social functions. Anger aims to change someone’s actions or dispositions, but contempt does not. On the contrary, contempt aims to exclude people from one’s social surroundings, to put physical and psychological distance between us and them. This exclusion or withdrawal can have different rationales; one might think that there is no way of changing or influencing X, or one may simply not want to put in the effort required to change X (Fisher and Roseman 2007).^15^ This is not to suggest that all expressions of contempt are appropriate. Just as one can fear too much, so one can contemn too much, which would constitute an instance of the vice of excess and in many cases an expression of closed-mindedness or bad taste.^16^ And just as it is difficult if not impossible to spell out in advance and in general the precise conditions under which fear is appropriate, so it is difficult if not impossible to spell out in advance and in general the precise conditions under which contempt is appropriate. We suggest that contempt is felt when one perceives a violation of a standard or norm or a way of behaving or being that one would expect from people. Contempt thus helps us to recognize and signal perceived failure, and when it is combined with laughter and amusement, it may help us to recognize the violation as benign, inhibiting the emotion of contempt when it is excessively strong and therefore vicious.

How does a sense of humor operate in such cases? When someone perceives a violation of a norm or standard by X and believes that they are not able (or willing) to change X’s ways, they feel contempt for X. We argue that, especially in those cases in which people feel a certain powerlessness to change someone or something, a sense of humor is of great value. As explained above, contempt motivates us to withdraw from the object of contempt. This is, however, sometimes impossible and most of the time does not change the despised person or thing. This impossibility follows from the circumstance that often the desired exclusion of a person or group from one’s social surrounding would imply socially excluding oneself from one’s own social setting, e.g., at work, in school, in the family, in a friendship circle, or more generally in the community or polity one lives in. And in cases of self-contempt, one obviously cannot separate from oneself. We think that in such cases a sense of humor can be helpful in dealing with one’s contempt, and that it can be a salutary way of expressing and directing one’s contempt. This is because we can transform our perception of our contempt towards an object into something acceptable at least for that moment (think of Sarah Cooper’s lipsyncs, which didn’t remove Trump from office but did help her and her audience to cope with the fact that he remained in office).

If this is on the right track, then having a sense of humor enables one and one’s audience to take someone or something less seriously than they otherwise would, reducing the emotion of contempt when it would be damaging to oneself. Humor makes it possible to laugh at people who are overly self-important or self-involved — including oneself when one is being overly self-important or self-involved.^17^ And because laughter can be infectious, a sense of humor may be especially valuable when people violate norms. For instance, much political satire targets a leader or regime that takes itself too seriously and needs to be taken down a notch or three. Likewise, much religious satire targets religious hierarchies or hierarchs who embody an unwarranted, holier-than-thou attitude. This is not to suggest that all political satire is appropriate or virtuous. A lot of political satire kicks down, but there are also clear cases in which it punches up in admirable ways.^18^

Sense of humor also serves the function of enhancing contempt in oneself and others when it is deficient. Moreover, as Roseman (2018; see also Dion 1979 and Myers 2013) argues, contempt binds together those who feel it for the same object. These facts suggest that contemptuous humor can be a powerful weapon against corrupt or authoritarian leaders and regimes, just as Zelenskiy remarked in the quotation above. Indeed, this political role of humor in eliciting and directing the contempt of others is presumably one of the reasons why political satire and other sorts of political humor are often forbidden in authoritarian states. *Lèse-majesté* laws have been used the world over to forbid mockery of kings, queens, and their inbred families. More recently, political satire has been used by Egyptian dissidents (and punished by the state) during the Arab Spring (Helmy and Frerichs 2013). And in the capitalist world, authoritarian workplaces helmed by petty tyrants may be just as liable as kings to punish contemptuous humor about their pretentions and self-importance. For instance, in 2010, Dawnmarie Souza was fired from her job over ridicule that she directed at her boss in a Facebook post (Greenhouse 2010).^19^

In the case of amusement, humor brings with it the positive affect that people often desperately need when it is hardest to laugh. In the case of contempt, humor simultaneously spreads contempt among those who are under the thumb of various powerful people and institutions, and portrays those powerful people and institutions as less important and less worthy than they might otherwise appear. Humorous contempt may also help people to cope with the inevitable suffering that accompanies human finitude, such as the pains of aging and ill health. It’s thus clear that the inhibition or enhancement of contempt by a sense of humor can be fitting. To the extent that someone is disposed to enhance and inhibit contempt that is fitting in this way, their sense of humor is a candidate for being a virtue.

### Trust

The political and more broadly social function of a sense of humor brings us to the third emotion that it enhances and inhibits according to appropriateness: trust. For our purposes here, trust can be both a practical and an epistemic emotion. It involves positive affect towards another agent on whose practical or epistemic agency one depends. It can be hard to know whom one can trust with what, and fraught political and employment situations amplify both the need for trusted partners and the cost of placing one’s trust in the wrong person. For example, consider the scene in *The Lives of Others* in which a Stasi official tells a joke about Erich Honecker, the party chairman: Honecker greets the sun by saying, “Good morning!” and the sun replies subserviently. But when Honecker greets the sun in the evening, it sneers, “Screw you, I’m in the West now!” Unfortunately for the joker, one of his audience members does not take kindly to the joke, requests his name and identification number, and has him demoted.

A sense of humor can help people to direct trust appropriately, eliciting it when appropriate and inhibiting when inappropriate. We understand trust as the emotion one directs towards another person when one expects them to exercise their competence in response to recognition of one’s dependence on them (Jones 2012). Trust can be misplaced, therefore, if it is directed at someone who either lacks competence in the relevant domain or is not responsive to the dependence of the trustor. While someone’s sense of humor only sometimes indicates their competence (e.g., when it presupposes certain kinds of expertise or background knowledge), it often does reveal what they do and don’t value. This is in part because humor often takes us by surprise, and laughter is both hard to suppress and hard to convincingly mimic, making it a reliable signal (Gervais and Wilson 2005; Owren and Bachorowski 2001, 2003). What someone is and is not disposed to be amused by is thus a defeasible shortcut to their values and expectations: what they do or don’t care about, what they find surprising or unsurprising, what counts as sacred or profane from their evaluative perspective. It’s for these reasons that we tend to feel that we can trust those who laugh when we laugh (and, just as importantly, don’t laugh when we don’t laugh). In this way, a sense of humor can play a crucial role in eliciting and directing trust (and distrust).^20^

If this is on the right track, one’s sense of humor provides evidence of the sorts of dependencies to which one would be responsive. A sense of humor’s role in enhancing and inhibiting trust is structurally different from its role in enhancing and inhibiting amusement. In the case of amusement, the humorous person enhances and inhibits others’ amusement at the object of their own amusement. By contrast, in the case of trust, the humorous person enhances and inhibits trust in themselves. If I make a joke about X, I aim to get you to be amused by X but to trust in *me* (and perhaps also in others who find the joke amusing). A sense of humor is therefore a mechanism that signals one’s trustworthiness to potential partners, making it a component of what Jones (2012) calls *rich* trustworthiness. On Jones’s account, someone is richly trustworthy with respect to a potential partner to the extent that he not only would prove trustworthy if depended upon but also reliably signals the ways in which he is (and is not) trustworthy to the potential partner. In other words, someone is richly trustworthy with respect to a potential partner if and only if he signals that he can be trusted when and only when he in fact can be trusted. We revisit the mechanics of humor and trust below in section 4.2, where we consider the function of humor in fostering connection between people.

### Hope

The final emotion that we wish to connect to a virtuous sense of humor is hope. Like amusement, contempt, and trust, hope can be enhanced and inhibited by certain types of humor. Hope is typically future-directed. One might hope that a certain event will take place, that a particular outcome will obtain, or that a particular action will be taken. Alternatively, one could have hopes about either the present or the past if one hopes to find out that a certain event has already taken place, that a particular outcome obtained, or that a particular action was taken. In either case, hope involves uncertainty — either about the future or about aspects of the past or present that are currently unknown. In addition, hope involves attributing positive value to the uncertain-but-hoped-for event or outcome. People don’t hope for things that they don’t care about, and they don’t hope for things that they wish wouldn’t happen.^21^

Our suggestion is that a sense of humor — including a dark sense of humor — may help to conjure up hope when it is desperately needed. One of the debilitating aspects of dark times is that they can leave us bitter and hopeless (Stockdale 2021). In this context, despair may sometimes involve a failure of imagination. Dark humor in dark times counteracts this lack of imagination.^22^ In particular, we contend that dark humor can be helpful because engaging in expressions of humor involves adopting an affective and evaluative perspective. Sometimes, perhaps most of the time, the adopted perspective is merely grounded in the values and sentiments that the humorist held previously. However, in some cases people adopt — if only briefly — a different affective and evaluative perspective from the one they typically occupy. When this happens, a perceived malign violation may appear as or be transmuted into a benign one. This is often the case in expressions of self-deprecating and dark humor.

As we argued above, when someone engages in dark humor about themselves, they make light of their own suffering; they express amusement at and contempt toward it. In so doing, they adopt an affective and evaluative perspective from which their own woes are less important or worthy of note than they might otherwise appear from the inside. This is the perspective we sometimes adopt towards long-past tribulations and long-overcome challenges, what we called above the perspective of the Spinozan *sub specie aeternitatis* or the laughing Epicurean gods. Monty Python and Mel Brooks were able to make jokes about the Spanish Inquisition in part because Tomás de Torquemada had been dead for more than five centuries when they were filming. But the same kind of psychological distancing can be temporarily adopted even in the thick of things, as the example of Zelenskiy above suggests. Indeed, there are even documented cases of hope-nourishing dark humor in Nazi concentration camps and death camps. For instance, Feig (1979; quoted in Morreall 2009, p. 123) recounts the words of Rabbi Erich Weiner, who was a leader of prisoners in one of the camps. These prisoners would regularly stage cabaret shows satirizing the Nazis and Hitler. According to Weiner, these shows “strengthened their will to survive as well as infused their power to resist.” Even in Dachau, the prisoners staged lampoons of Hitler that were attended by members of the SS. One survivor of the camp later said that many of the prisoners

who sat behind the rows of the SS each night and laughed with a full heart, didn’t experience the day of freedom. But most among them took from this demonstration strength to endure their situation…. They had the certainty, as they lay that night on their wooden bunks: We have done something that gives strength to our comrades. We have made the Nazis look ridiculous. (Migdal 1986; quoted in Morreall 2009, p. 123)

Outside the camps, anti-fascist humor was common both before and during the war. According to Morreall (2009, p. 124), one joke went as follows:

Goebbels was touring German schools and asked the students to call out patriotic sayings:

“Heil Hitler,” shouted one child.

“Very good,” said Goebbels.

“*Deutschland über alles*,” another called out.

“Excellent. How about a stronger slogan?”

“Our people shall live forever,” the little boy said.

“Wonderful,” exclaimed Goebbels. “What is your name, young man?”

“Israel Goldberg.”

This sort of humor can, we contend, sometimes (though of course only sometimes) foster the hope and solidarity needed to remain agentic in dark times. And it may do so in a way that motivates collective action the fruits of which the hoper never lives to enjoy.

If these speculations are on the right track, then in bouts of dark humor, we project ourselves into an alternative point of view, which can be a potential future, from which our own sufferings will be the sort of thing we or others like us can laugh about. In so doing, we make it possible to imagine such a future, and we may even make it possible to envision how we might work, Moses-like, towards bringing about such a world even if it is not a world that we will live to see. Dark humor about climate change may serve as another example (e.g., the meme currently going around pointing out that while this summer might be the hottest of your life, it’s also the coldest of the rest of your life). If this is on the right track, then a sense of humor has a role to play in expanding our imaginative capacities.

In some cases, though, hope can be excessive, especially if it is directed at future events that are unrealistic, or the pursuit of which is damaging to the hoper or others. Romantic interest is a common context for the kind of excessive hope, which can be destructive. People who stubbornly hope for requited affections from their beloved, even after being rejected multiple times, often let their hope get in the way of their own recovery from heartbreak. Excessive and inappropriate hope can also fuel workaholism, gambling addiction, and toxic positivity.^23^ A sense of humor can help to inhibit such destructive hope in oneself and others, for instance, through mockery of toxic positivity. Cliché hopeful positivity catchphrases such as “when life gives you lemons, make lemonade” have been met with retorts like “when life gives you lemons, squeeze them in people’s eyes.” We are also inclined to ridicule unwarranted and destructive hopes in general. We might dissolve excessive hope by joking about the naivety of entrepreneurs who think electric cars or colonies of indentured servants on Mars will solve the climate crisis. Thus, dark humor may also play a positive role in providing a reality-check when it is needed. But when it is cynically directed at activism that might otherwise be effective, in right-wing mockery of virtue signaling and moral grandstanding (Tosi and Warmke 2020), it errs in the opposite direction.

## The Functions of a Sense of Humor, and Their Contexts

Above, we canvassed four emotions that a sense of humor helps regulate by enhancing when they are deficient and inhibiting when they are excessive. We now associate these emotions with the functions served by a sense of humor and the contexts in which those functions are most relevant. These functions have already been gestured at, but we address them more explicitly here. In so doing, we follow Foot’s (1997, p. 3) approach to the virtues, according to which “virtues are in general beneficial characteristics, and indeed ones that a human being needs to have, for his own sake and that of his fellows.” According to Foot, we need a range of virtues to help overcome challenges and take advantage of opportunities that are likely to crop up in our lives. Almost everyone faces some fearsome threats, which is why almost everyone needs at least a modicum of courage. In societies beset by inevitable or human-made shortages of resources, almost everyone faces opportunities to help others who are less well off, which is why almost everyone needs at least a modicum of generosity. We contend that having a sense of humor is as universally-needed as other, more-traditional virtues. In this section, we argue that this is the case because having a sense of humor answers to four universal human needs: coping, connection, capability, and criticism. The contexts in which these needs arise include hardship, fostering relationships, collective action, and existential reflection.

### Coping

Some day in the coming weeks or decades, you will die. In the meantime, you will probably experience pain, suffering, anguish, and self-doubt. Worst of all, it’s likely that many or even most of the people you love will die before you do. Life is hard that way. This human universal means that we need ways to cope with suffering — both our own and that of our loved ones. Having a sense of humor helps us cope in several ways. First and foremost, a sense of humor elicits and directs amusement, and a dark sense of humor elicits and directs humor in the context of suffering, fragility, finitude, and evil. Empirical research suggests that humor and laughter have therapeutic value in hospitals, long-term care facilities, hospice settings, and nursing homes (Aultman 2009; Wilkins and Eisenbraun 2009; Mora-Ripoll 2010).

Beyond momentary hardships, a sense of humor seems to be especially valuable in coping with one’s own imperfections (especially malleable imperfections) because it elicits and directs contempt at them. Someone who is able to laugh at their own imperfections is also, sometimes, able to see those imperfections as unimportant or trivial (Alfano 2019, chapter 9). This makes it possible to abandon them, to change, to become a different and perhaps more worthy and interesting person. A sense of humor can make serious things less serious — or at least make them seem less serious than they appear in times of hardship.

Finally, a sense of humor can help people to cope with hard existential truths. One of the better jokes in Freud’s *Jokes and Their Relation to the Unconscious* (1905 / 1974) is an updated version of the wisdom of Silenus. In the myth, the satyr Silenus tells King Midas that the best thing for a human is never to have been born, and that the second best is to die soon. Freud recounts a joke popular in his own circles that goes, “Life is so terrible, it would have been better not to have been born. Who is so lucky? Not one in a hundred thousand!”

### Connection

Humans are a hyper-social species. We need social connections to survive and thrive. Such connections inevitably involve trust. As we argued above, one’s sense of humor is a royal road to establishing trust with others who share one’s values, concerns, and expectations. It’s possible to use one’s sense of humor as a kind of divining rod or touchstone to these values, concerns, and expectations. Indeed, one could aim to establish a community by eliciting laughter that only those who share one’s expectations and values can muster. Moreover, isolation and alienation are among the hardest things for people to cope with, so when one’s sense of humor elicits and directs trust, it also helps one to cope.

In this context, we understand connection to refer not to the maximization but to the fine-tuning of social relations. What we want is not to have as many trusting relationships as possible but to have trusting relationships with *enough* and *the right* people. In general, that will mean forming and maintaining relationships with people whose values and concerns match our own or those we aspire to. Exercising one’s sense of humor is an important, if not essential, way to form and maintain such connections. Laughter strengthens bonds between people. When we laugh together, we strengthen our sense of community (Dezecache & Dunbar 2012; Curry & Dunbar 2013; Gordon 2014). This is in part because laughing together is pleasant, but there’s more to the story. Laughing together is also an indication of shared mindset and values. And because laughter typically arises spontaneously, laughter is a difficult-to-fake, difficult-to-stifle expression of what one does and does not value.

To elucidate this point, let us return to the connection between a sense of humor and trust. Consider Alfano’s (2019) tetrapartite framework for thinking about the social dimensions of humor. Alfano argues that we should distinguish at least four different roles in a typical episode of humor. Schematically, W laughs with X (but not with Y) at Z. The person in the W-role is the producer of humor, the one who cracks wise. The person in the X-role is the appreciator of humor, the one who is amused by the humor. The person in the Y-role is the outsider, who either doesn’t get the joke or considers it in bad taste. And the thing or person in the Z-role is the object of humor, the thing or person who serves as the butt of the joke. More than one person can play a given role; for example, there might be multiple audiences to a joke, meaning that multiple people play the X-role. In addition, the same person may play multiple roles. For instance, in self-deprecating humor, the same person plays both the W-role and the Z-role, and people who successfully employ self-deprecating humor may garner others’ trust in domains where they don’t mock their own abilities, since someone who is willing to admit flaws in one domain is less likely to claim abilities they lack in other domains.

**Figure 1:**
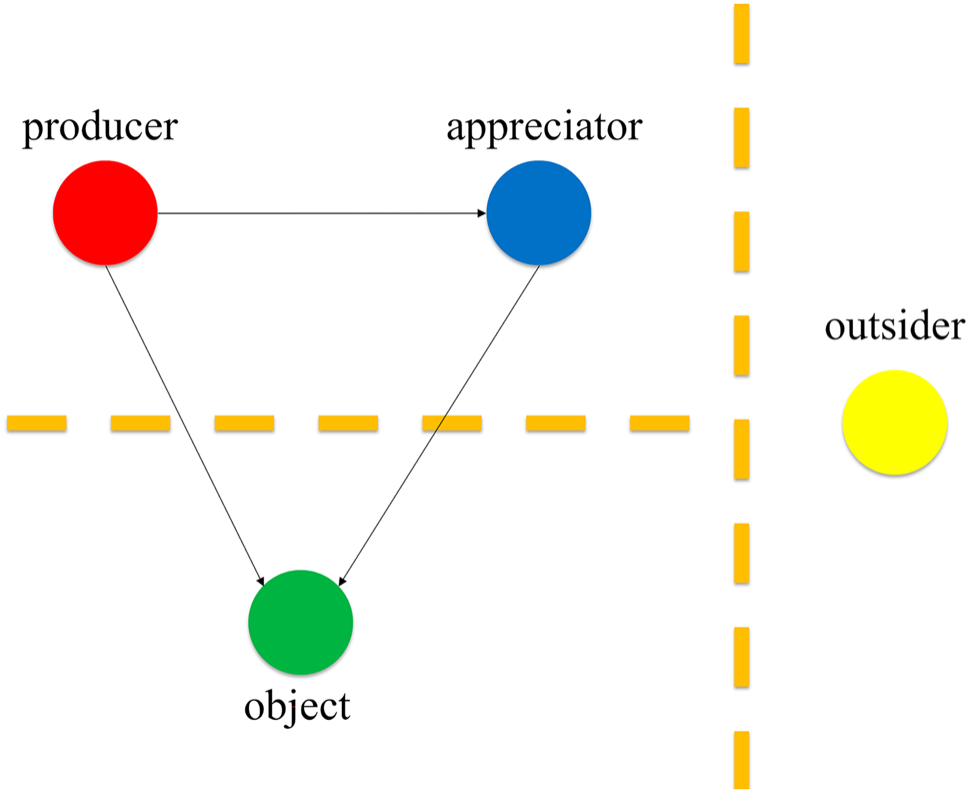
the geometry of humor

In this framework, W and X join together in a community of laughter that looks down on Z, and from which Y is excluded, as pictured in Figure 1.^24^

We believe that these distinctions are helpful for understanding why a sense of humor is highly valued and what it consists in. In this framework, making a joke is an invitation to join in a community of laughter. How someone responds to that invitation is expressive of their own sense of humor. If they respond as an appreciator, they signal shared values with the producer, which in turn may make them more willing to put their trust in the producer. If, by contrast, they respond as an outsider, they signal that their values and the values of the producer are disjoint, or that they lack the cultural and expert knowledge required to understand the humor. Having a discriminating sense of humor enables those in the Y role to demonstrate, for instance, that they do not appreciate racist and misogynistic humor, and thus that they do not embody racist and misogynistic values. This is not meant to imply that only racists laugh at racist jokes or that only misogynists laugh at misogynistic jokes. The point is about general tendencies. And reflective people will tend to notice and respond appropriately when they find themselves amused by offensive humor that expresses values that they do not on reflection endorse.^25^

In this way, both the production and the appreciation (or not, on reflection) of humor help people sort themselves into like-minded and like-valued communities, into groups that can reasonably trust their fellow group members. Moreover, someone’s ability to cope with being the object of humor, to laugh at themselves, indicates the extent to which they take themselves overly seriously or suffer from vanity. Someone who is completely unable to handle being the butt of a joke (even a good-natured jest by a friend) betrays inflated self-concern or a fragile ego. By contrast, someone who laughs at every joke directed at them, no matter how mean-spirited, confesses to a pathological lack of self-respect. If these reflections are on the right track, then a virtuous sense of humor is multifaceted in the sense that it enables one to play the producer, appreciator, outsider, and object roles well. One way to interpret this point is to say that there are actually four discrete senses of humor — one for each role. Alternatively, we might say that a sense of humor is a multi-track disposition, with modules pertaining to each role. Someone possesses a virtuous sense of humor to the extent that they embody and integrate each of the modules.^26^ It may be that each of these dispositions is to some extent distinct, though we suspect that they tend to be correlated empirically.

At the same time, the contempt elicited and directed by one’s sense of humor enables one to sever toxic connections. Thus, humor helps both to establish social connections and to cut them, a mildly paradoxical pair of functions that makes sense when we think of humor not as maximizing connection but as curating it.^27^ Many such connections are to those with whom we used to have something in common but who no longer share our values, such as family members, people from our hometowns, and others to whom we are connected by un-elective affinities.^28^ Alfano (2019, chapter 10) argues that Nietzsche’s notion of *solitude* (*Einsamkeit*) answers to the need to sever such un-elective affinities. Nietzschean solitude is the disposition to adopt a critical, contemptuous attitude towards one’s ingroups, especially ingroups that one never voluntarily joined. A sense of humor is crucial to such self-criticism at the collective level, as it makes possible the distanced perspective needed to think and speak ill of one’s ingroup.

### Capability

We understand capability as the power or ability to do or prevent something. One of the most important capabilities is the power or ability to do or prevent something in concert with others. That is to say, collective action and agency are vitally important parts of our lives and societies. However, in order to engage in collective action and agency, we need at least a modicum of assurance that the other people involved will play their part and live up to expectations. This is where the trust-enhancing and trust-inhibiting aspects of a sense of humor come into focus. As we argued above, one’s sense of humor can help one discover both those one can reasonably trust and those one cannot reasonably trust. In so doing, a sense of humor makes it possible to form communities of like-minded and like-valued individuals who both do and should trust one another (assuming the relevant competencies are also in place). Especially when the collective action to be undertaken is fraught or dangerous (e.g., engaging in a labor strike, fighting a bushfire, protesting police brutality, military combat), it is absolutely essential that all parties not only trust one another but are assured that their trust is well-placed. Having a history of shared humor is one strong indicator that such trust is indeed well-placed. Perhaps this explains why humor is a common collective coping mechanism among both firefighters and warfighters (Sliter et al. 2013; Bizi et al. 1988).

In addition, the hope-enhancing and hope-inhibiting power of a sense of humor facilitates capability and collective agency by making it possible for a group — not just an individual — to envision, imagine, and work towards a desirable future. As we argued above, hopelessness is sometimes engendered by a lack of imagination, and a sense of humor helps one to imagine future points of view from which current woes are unimportant or less debilitating than they currently seem. This holds not just for individual hope but, perhaps even more so, for collective hope. In many cases, the sorts of change we would like to see can only be brought about through collective action and social coordination. Laughing with others can sometimes make it possible to envision a future in which one works together with them to effect such change. At the same time, laughing off unrealistic hopes, and inhibiting excessive hope in bad ideas, can also help to keep collective action on track.

We suggest that a sense of humor is an executive virtue, much like courage and self-control. Executive virtues are dispositions that make someone a more effective agent, regardless of their goals. Notoriously, such virtues can be paired with substantive vices and make one more effective in the pursuit of evil ends. Like most powers, they can be turned to good but also to ill.^29^ One thing that distinguishes a sense of humor from self-control is that it is an executive virtue relative to collective agency rather than only to individual agency. Like other executive virtues, a sense of humor is powerful, which can make it dangerous. However, just as we would not wish to live in a world full of cowards with poor impulse-control, so we would not wish to live in a world full of humorless people.

### Criticism

The final function we envisage for a virtuous sense of humor is criticism. Because a sense of humor is able to express, elicit, and direct the emotion of contempt, it fosters critical engagement with people, things, and institutions that might otherwise be immune to criticism. As Nietzsche puts it in *Thus Spoke Zarathustra*, “Not by wrath does one kill, but by laughing” (Book 1, “On reading and writing”). In political and employment contexts, humor expresses contempt for people in power who aren’t really as powerful, clever, and wise as they take themselves to be. However, expressing such humor can be dangerous, so it may need to be accompanied by courage, prudence, and a concern for justice. We further note that a sense of humor can go wrong if it systematically directs contempt not to the rich and powerful but downward, towards oppressed and underprivileged groups. This is a point that the comedian George Carlin famously made during an interview with Larry King in 1990.^30^

Beyond criticizing others, a sense of humor plays a role in self-criticism, including both individual and collective self-criticism. Alfano (2019, chapters 9) argues for the importance of self-criticism and self-contempt.^31^ It can be hard to see and appreciate one’s own flaws, but when one is able to laugh at oneself, this becomes possible. Moreover, because such self-contempt is also accompanied by amusement, the bitter pill becomes a bit easier to swallow. The positive affect induced by humor breaks down psychological defenses that might otherwise interfere with recognition of one’s own flaws and imperfections.

As we mentioned above, Alfano (2019, chapter 10) goes further, arguing that Nietzsche celebrates a virtue that he calls ‘solitude’ (*Einsamkeit*), which expresses, elicits, and directs *collective* self-contempt and self-criticism. In other words, solitude is less about contempt for the ‘I’ and more about contempt for the ‘we’, for one’s ingroup. Such collective self-contempt is valuable in much the same way that individual self-contempt is: it enables people to see the flaws and imperfections of their ingroup, which might otherwise be protected by various psychological and socio-cultural defenses. Furthermore, to the extent that such humor succeeds in eliciting amusement as well as contempt, it makes the criticism easier to swallow.

If these reflections are on the right track, then a sense of humor is a valuable way to engage in criticism of others, of oneself, and of one’s ingroup. In the latter two contexts, a sense of humor may foster self-improvement.

## Conclusion

In this paper, we argued that a sense of humor is a virtue because it answers to universal human needs. A sense of humor helps people cope with hardship. It helps them connect socially to others, bonding selectively with those who share their values. It supports capability, especially when it comes to collective action and agency. And it expresses and makes palatable criticism, both of oneself and of others. A sense of humor is able to serve these functions because it has the power to enhance or inhibit the emotions of amusement, contempt, trust, and hope when they are deficient or excessive. When it succeeds in expressing, eliciting, and directing these emotions in fitting ways, it should be considered a virtue. Moreover, at least when it serves the purpose of fostering capability, a sense of humor is an executive virtue. In future research, it would be worthwhile to examine empirically the extent to which a sense of humor opens up people’s imaginative capacities. In this paper, we hope to have made a plausible case that it *sometimes* does so, but we have not established how often, how effectively, or for how long. In addition, it would be worthwhile to reflect further on the functions and dysfunctions of a sense of humor. For example, we only briefly touched upon problematic examples such as racist and sexist humor – not to mention humor that’s used to bully others; these clearly deserve further attention.^32^

## Footnotes

1 Thanks to Ritsaart Reimann, Ellen Fridland, Matthew Stichter, Tad Zawidzki, Quinn Hiroshi Gibson, and Bana Bashour for comments on a draft of this manuscript. This project/publication was made possible through the support of grants from the John Templeton Foundation (#61378) and the Australian Research Council (DP190101507). The opinions expressed in this publication are those of the author(s) and do not necessarily reflect the views of the John Templeton Foundation or the Australian Research Council.

2 Url = <https://www.wbur.org/hereandnow/2020/06/08/sarah-cooper-comedy-trump>, accessed 24 June 2020.

3 Url = <https://www.theatlantic.com/international/archive/2022/04/zelensky-kyiv-russia-war-ukrainian-survival-interview/629570/>, accessed 23 April 2022.

4 See Shoemaker (2018) for further reflection on the relationship between humor and psychological impairments.

5 The distinction between the dispositions to tell versus take a joke (as well as to appreciate a joke made at another’s expense) is explored more systematically in section 3.3 below.

6 See Southam (2005) and Semrud-Clikeman and Glass (2010) for developmental and clinical approaches to the acquisition of a sense of humor in children.

7 Note that, while the Aristotelean framework remains dominant in contemporary virtue theory, we needn’t commit to an Aristotelian framework to understand what it would take to have a virtuous sense of humor. For instance, in a recent monograph, Alfano (2019, chapter 9) advances the hypothesis that having a sense of humor is a virtue in the Nietzschean framework. For more on Nietzsche and humor, see Ansell-Pearson (1994), Gordon (2016), Higgins (1994, 2000), Lippitt (1992), and Weeks (2004).

8 Note that these may not be the only emotions that can be connected with a sense of humor. However, we think that they are the most important ones when it comes to a virtuous sense of humor.

9 For an empirical distinction between mirth, which is closely related to amusement, and various other positive emotions such as elevation, see Strohminger et al. (2011). For more on the moral psychology of amusement, see Robinson (2021).

10 Other popular theories of humor and draw some connection to emotional expressions include the superiority theory, the incongruity theory, and the relief theory. For an overview, see Olin (2016, 2022).

11 For more on fitting emotions, see D’Arms and Jacobson (2000) and Jacobson (2011).

12 See, for example, url = <https://www.inkedmag.com/culture/check-womans-creative-behind-ear-mute-tattoo>, accessed 24 June 2020.

13 Note that we are not suggesting that the emotion of contempt is itself a virtue. That would be a category error. Nor are we suggesting that every expression of contempt is virtuous. Rather, our claim is that the disposition to feel contempt in the right way is a virtue. This is in keeping with the Aristotelean analysis of the relationship between virtues and emotions more broadly. For example, fear is not a virtue, but the disposition to feel fear when and only when it is appropriate is a virtue. Thanks to an anonymous referee for pressing us on this point.

14 For more on the nature and moral psychology of contempt, see Bell (2013) and Mason (2018).

15 For more on the role played by anger in partner control and the role played by contempt in partner choice, see Martin and Cushman (2015).

16 Thanks to an anonymous referee for pressing us on this point.

17 Like amusement, contempt can be higher-order. For example, Bell (2013) argues that contempt for others’ contempt is sometimes a fitting attitude, and Alfano (2018, 2019, chapters 8-9) shows that Nietzsche placed great value on the capacity for *spernere se sperni* (contempt for others’ contempt).

18 Thanks to an anonymous referee for pressing us on this point.

19 For further illustrations, see Teitel (2012), who canvasses several other cases in both the United States and Canada.

20 See Schloss (2007) for a psychological perspective on laughter as a signaling mechanism.

21 For more on the nature, function, and value of hope, see Martin (2013), Blöser and Stahl (2019), and Stockdale (2021).

22 Of course, sometimes bitterness, hopelessness, or despair is entirely appropriate. Our point is that despair *sometimes* gets it wrong, not that it always does so.

23 For a discussion of toxic positivity, see Upadhyay et al. (2022).

24 See Freud (1905 / 1974) for some related remarks in the context of smutty humor.

25 Thanks to an anonymous referee for pressing us on this point.

26 See Adams (2006) for a similar modular treatment of the virtue of courage.

27 For more on this paradox of humor, see Olin (2016).

28 This is not to suggest that all severing of un-elective affinities is valuable. But surely sometimes the best thing to do is to cut off a relationship, and the decision should be guided by practical wisdom. If this is right, then the contempt summoned by a sense of humor may be just the thing to help someone move on from an unsalvageable relationship. Thanks to an anonymous referee for pressing us on this point.

29 This perhaps explains the double-edged nature of sexist, racist, and otherwise prejudiced humor (Abramson 2014; Bergmann 1986; Smuts 2007). See also Morreall (2009, p. 105).

30 See https://www.youtube.com/watch?v=R7Xkey41AYQ. See also Healy (2016).

31 See also Morreall (1983, p. 123).

32 For some recent work on ethically problematic humor, see Gout (1998), Jacobson (1997), Morgan & DiFranco (2021), and Nash (2021).
